# Bridging the post-discharge management gap in venous thromboembolism: linking adherence patterns to persistent hypercoagulability

**DOI:** 10.3389/fcvm.2026.1773167

**Published:** 2026-02-17

**Authors:** Yingxin Wang, Xiaping Zhang

**Affiliations:** 1 Department of Vascular Surgery, The Second Xiangya Hospital of Central South University, Changsha, China; 2 Department of Clinical Nursing, The Second Xiangya Hospital of Central South University, Changsha, China

**Keywords:** biomarker monitoring, chronic disease management, D-dimer, hypercoagulability, medication adherence, post-discharge care, smoking cessation, venous thromboembolism

## Abstract

**Objective:**

This observational study investigates the relationship between post-discharge adherence to venous thromboembolism (VTE) preventive measures (including anticoagulant therapy, compression therapy, and follow-up attendance) and the resolution of hypercoagulability, as assessed through serial D-dimer measurements. It is important to note that clinical recurrence outcomes were not directly measured in this study; the primary endpoint was biomarker trajectory.

**Methods:**

Conducted at a tertiary vascular surgery department in Changsha, China, between April and September 2024, this prospective cohort study enrolled 110 patients with confirmed VTE diagnoses. Participants were stratified into three adherence categories based on their post-discharge compliance with prescribed anticoagulation and compression therapies using objective criteria of treatment completion and self-reported behavior: complete adherence (Group A, *n* = 40), partial adherence (Group B, *n* = 44), and non-adherence (Group C, *n* = 26). We measured D-dimer levels at diagnosis and at the 90-day follow-up, calculating absolute changes (ΔD-dimer = D-dimerfollow-up—D-dimerbaseline) to evaluate resolution of hypercoagulability. Statistical analyses employed ANOVA for group comparisons and multivariable linear regression to identify predictors of persistent D-dimer elevation.

**Results:**

Only 38.4% of patients maintained consistent use of elastic stockings throughout the 90-day follow-up period, with adherence declining most substantially between 30 and 60 days post-discharge. D-dimer trajectories revealed marked intergroup differences: patients in the non-adherence group demonstrated minimal reduction in D-dimer levels (Δ −0.52 μg/mL; 95% CI: −1.46–0.42), whereas those in the complete adherence group showed substantial decreases (Δ −4.87 μg/mL; 95% CI: −5.92 to −3.82; *p* < 0.001). Current smoking emerged as the strongest independent predictor of sustained D-dimer elevation (*β* = 6.121, *p* < 0.001), followed by non-adherence status (*β* = 4.991, *p* < 0.001). The period around 60 days post-discharge represented a critical “adherence cliff” that coincides with established windows of elevated recurrence risk, though this link is inferential based on biomarker data rather than observed clinical events.

**Conclusion:**

Our findings establish a direct biological link between the post-discharge “management gap” in VTE care and persistent hypercoagulability. We propose reframing VTE as a chronic high-risk condition necessitating structured long-term management, with particular emphasis on interventions targeting the 60-day transition period and smoking cessation as high-yield strategies. These insights advocate for a paradigm shift from acute treatment to sustained risk control in VTE management.

## Highlights

Post-discharge adherence to VTE preventive measures declines substantially, with only 38.4% maintaining consistent elastic stocking use at 90 days.
Non-adherent patients exhibit minimal D-dimer reduction (Δ −0.52 μg/mL) compared to substantial decreases in adherent patients (Δ −4.87 μg/mL).
Current smoking represents the strongest predictor of sustained hypercoagulability (*β* = 6.121, *p* < 0.001).
The 60-day post-discharge period constitutes a critical “adherence cliff” requiring targeted interventionxac b aaxc strategies.
D-dimer monitoring provides objective evidence connecting clinical management practices to biological risk profiles.

## 1 Introduction

Venous thromboembolism (VTE) imposes a substantial global health burden, characterized by significant morbidity, mortality, and healthcare expenditures (1). Despite therapeutic advances in prevention and management, recurrence rates remain concerning, with approximately 10%–30% of patients experiencing recurrent events within 5–10 years following their initial episode (2, 3). This persistent risk challenges the conventional perception of VTE as an acute, self-limited condition and suggests underlying chronic pathophysiological mechanisms that extend beyond the initial thrombotic event (4, 5).

The transition from inpatient care to community-based management represents a particularly vulnerable phase for VTE patients, often marked by declining adherence to prescribed therapies and diminished clinical oversight (6, 7). This “post-discharge management gap” has been documented across diverse healthcare systems, with studies indicating that up to 50% of patients discontinue anticoagulant therapy prematurely and compliance with compression therapy deteriorates significantly within the first three months (8, 9). This gap may be influenced by the pre-discharge preparation process, including patient education, discharge counseling, anticoagulation planning, and follow-up scheduling, which sets the stage for post-discharge self-management (10, 11). However, the biological consequences of this management gap remain incompletely characterized, particularly regarding its impact on sustained hypercoagulability. Furthermore, this study focuses on biomarker outcomes (D-dimer trajectories) as a surrogate for biological risk; clinical recurrence events were not directly measured.

D-dimer, a specific fibrin degradation product, has emerged as a valuable biomarker for assessing hypercoagulability and predicting VTE recurrence risk (12, 13). Prospective investigations have demonstrated that persistently elevated D-dimer levels following anticoagulation cessation are associated with significantly increased recurrence risk (14, 15). Serial D-dimer monitoring post-anticoagulation is a strategy of ongoing research interest for risk stratification (16). Current guidelines incorporate D-dimer assessment combined with clinical risk stratification (using tools such as the DASH score or HERDOO2 rule) to identify patients who may safely discontinue anticoagulation (17, 18). The change in D-dimer levels (ΔD-dimer), calculated as the difference between follow-up and baseline measurements (ΔD-dimer = D-dimerfollow-up—D-dimerbaseline), serves as a quantifiable measure of hypercoagulability resolution (19). Nevertheless, the relationship between adherence to preventive measures and longitudinal D-dimer trajectories remains inadequately explored.

This study addresses this critical evidence gap by examining the association between post-discharge adherence to VTE preventive measures (encompassing anticoagulation, compression therapy, and follow-up) and serial changes in D-dimer levels. By stratifying patients according to adherence patterns and correlating these with corresponding biomarker responses, we aimed to generate evidence-based insights to optimize long-term VTE management strategies in line with contemporary guideline updates emphasizing structured follow-up (20).

## 2 Methods

### 2.1 Study design and setting

This prospective observational cohort study was conducted in the Department of Vascular Surgery at a tertiary referral center (The Second Xiangya Hospital of Central South University, Changsha, China) between April and September 2024. The study was designed to evaluate biomarker trajectories in relation to adherence behaviors over a 90-day post-discharge period.

### 2.2 Ethical considerations

The study protocol received approval from the Institutional Review Board of The Second Xiangya Hospital of Central South University (approval number Z0568-02) and was conducted in accordance with the ethical principles outlined in the Declaration of Helsinki (21). All participants provided written informed consent prior to enrollment. The study was conducted in accordance with Good Clinical Practice guidelines (22). Confidentiality was maintained through secure data management systems, and participants were informed of their right to withdraw at any time without affecting their ongoing medical care.

### 2.3 Study population

#### 2.3.1 Inclusion criteria

(1) Adult patients (≥18 years) with objectively confirmed acute VTE [deep vein thrombosis [DVT] and/or pulmonary embolism [PE]], with diagnostic confirmation adhering to established clinical guidelines (23).
(2) Planned to receive standard anticoagulation therapy (minimum duration 3 months) according to contemporary clinical guidelines (24).
(3) Capable of providing informed consent and participating in scheduled follow-up assessments.

#### 2.3.2 Exclusion criteria

(1) Life expectancy <3 months due to comorbid conditions.
(2) Contraindications to both anticoagulation and compression therapies.
(3) Concurrent participation in other interventional clinical trials.
(4) Inability to complete study questionnaires or comply with follow-up procedures.

### 2.4 Sample size considerations

A total of 110 participants were enrolled, comprising 54 males (49.1%) and 56 females (50.9%), with a mean age of 58.3 years (SD: 12.5). Based on preliminary data indicating an anticipated mean D-dimer difference of 3.5 μg/mL between adherence groups with a standard deviation of 4.0 μg/mL, we estimated that 26 participants per group would provide 80% statistical power to detect significant differences (*α* = 0.05, two-sided) (25). Accounting for an estimated 20% attrition rate, we targeted enrollment of 110 participants, consistent with sample size calculation methodologies for observational studies (26).

### 2.5 Adherence assessment and categorization

Adherence was evaluated through structured telephone interviews conducted at 30, 60, and 90 days post-discharge. Medication adherence was assessed using two objective criteria: (1) completion of the prescribed 90-day anticoagulation course, and (2) the absence of self-reported intentional medication omissions or dosage modifications. Compression therapy adherence was assessed based on patient-reported use of elastic stockings for ≥8 h daily.

Participants were categorized into three distinct groups based on predetermined criteria that align with established adherence classification frameworks (27):

#### 2.5.1 Group A (complete adherence)

(1) Completed the prescribed 90-day anticoagulation regimen without premature discontinuation and reported no intentional medication omissions or dosage modifications.
(2) Used compression stockings ≥8 h daily for ≥80% of days during the follow-up period, based on established compression therapy protocols (28).

#### 2.5.2 Group B (partial adherence)

(1) Completed either the 90-day anticoagulation course without intentional deviation OR maintained adequate compression stocking use (as defined above), but not both concurrently.
(2) Exhibited minor deviations from prescribed regimens (e.g., occasional unintentional forgetting or compression stocking use on 50%–79% of days), consistent with established partial adherence definitions (29).

#### 2.5.3 Group C (non-adherence)

(1) Discontinued anticoagulation therapy prematurely (<90 days) and/or reported intentional therapy discontinuation or frequent omissions.
(2) Used compression stockings for <50% of days during follow-up, or used for <80% of days combined with failure to complete anticoagulation.

The detailed scoring and operationalization criteria for adherence categorization are provided in Appendix 1.

### 2.6 Biomarker assessment

Venous blood samples for D-dimer quantification were collected at diagnosis (baseline) and at the 90-day follow-up using standardized phlebotomy procedures (30). All samples underwent processing within 2 h of collection and were analyzed using a standardized, high-sensitivity latex-enhanced immunoturbidimetric assay (STA-Liatest D-Di, Diagnostica Stago) with documented inter-assay coefficient of variation <5% (31). ΔD-dimer was calculated as the absolute difference between the 90-day follow-up and baseline measurements (ΔD-dimer = D-dimerfollow-up—D-dimerbaseline). The selection of D-dimer as a biomarker of hypercoagulability is supported by extensive validation in VTE management studies (19, 32).

### 2.7 Data collection

Data were systematically collected using a comprehensive case report form that captured:

#### 2.7.1 Demographic and clinical characteristics

Age, sex, body mass index (BMI), smoking status (categorized according to CDC definitions) (33), alcohol consumption patterns, comorbidities.

#### 2.7.2 VTE-specific factors

Anatomical location of thrombosis, provoking factors, Caprini risk score (34), previous VTE history.

#### 2.7.3 Treatment details

Initial and maintenance anticoagulant regimens, compression therapy prescriptions, treatment duration.

#### 2.7.4 Adherence metrics

Anticoagulation treatment completion (yes/no), self-reported intentional non-adherence behavior (yes/no), compression stocking utilization patterns, reasons for non-adherence categorized according to established frameworks (35).

#### 2.7.5 Laboratory parameters

Serial D-dimer measurements, complete blood count, renal and hepatic function tests.

### 2.8 Statistical analysis

Statistical analyses were performed using SPSS version 25.0 (IBM Corp.) and R version 4.2.0. Continuous variables were assessed for normality using Shapiro–Wilk tests and presented as mean ± standard deviation or median (interquartile range) as appropriate (36). Categorical variables were summarized as frequencies and percentages.

Intergroup comparisons employed one-way ANOVA with Tukey's *post-hoc* tests for continuous variables, while chi-square or Fisher's exact tests were used for categorical comparisons (37). Within-group changes in D-dimer levels were evaluated using paired *t*-tests or Wilcoxon signed-rank tests based on distributional characteristics (38).

Multivariable linear regression with backward elimination (retention threshold: *p* < 0.05) identified independent predictors of 90-day D-dimer levels. Variables demonstrating *p* < 0.10 in bivariate analyses were considered for inclusion in the regression model, consistent with established variable selection methods (39). Model assumptions were verified through residual analysis, and multicollinearity was assessed using variance inflation factors (VIF < 5 considered acceptable) (40).

All statistical tests were two-sided, with statistical significance defined as *p* < 0.05. Missing data were addressed using multiple imputation with five imputed datasets when missingness was <5%; cases with >5% missing data were excluded from the primary analysis (41).

## 3 Results

### 3.1 Baseline characteristics

The baseline characteristics of the study cohort are summarized in Table 1. The cohort had a balanced sex distribution (49.1% male). Most participants (58.1%) were classified as high or very high risk according to Caprini scores. Smoking prevalence was 34.5%, with significant variation across adherence categories (*p* = 0.016).

**Table 1:** Baseline characteristics stratified by adherence category.

Characteristic	Complete adherence (*n* = 40)	Partial adherence (*n* = 44)	Non- adherence (*n* = 26)	*p*-value
Demographics				
Age, years, mean (SD)	56.2 (12.4)	59.1 (11.8)	60.3 (13.2)	0.345
Male sex, *n* (%)	18 (45.0)	23 (52.3)	13 (50.0)	0.786
BMI, kg/m², mean (SD)	23.8 (3.1)	24.4 (2.9)	24.5 (3.3)	0.612
Risk factors				
Current smoking, *n* (%)	8 (20.0)	16 (36.4)	14 (53.8)	0.016*
Regular alcohol use, *n* (%)	12 (30.0)	17 (38.6)	9 (34.6)	0.702
≥2 Comorbid conditions, *n* (%)	22 (55.0)	28 (63.6)	18 (69.2)	0.495
Previous VTE history, *n* (%)	5 (12.5)	7 (15.9)	6 (23.1)	0.518
Clinical features				
Proximal DVT, *n* (%)	28 (70.0)	33 (75.0)	18 (69.2)	0.823
Pulmonary embolism, *n* (%)	4 (10.0)	6 (13.6)	4 (15.4)	0.791
Caprini score ≥5, *n* (%)	22 (55.0)	26 (59.1)	16 (61.5)	0.847
Laboratory parameters				
Baseline D-dimer, μg/mL, mean (SD)	7.21 (4.35)	7.68 (4.92)	7.85 (5.14)	0.831
Platelet count, ×10⁹/L, mean (SD)	245 (68)	238 (72)	252 (65)	0.692

* Statistical significance at *p* < 0.05.

### 3.2 Adherence trajectories

Post-discharge adherence exhibited progressive decline over time. While 88.5% of patients reported taking anticoagulants as prescribed at 30 days, this proportion decreased to 73.1% by 60 days and remained stable through 90 days. Compression stocking adherence demonstrated more pronounced deterioration: from 78.8% at 30 days to 57.7% at 60 days and only 38.4% at 90 days.

The period around 60 days post-discharge emerged as a critical transition point, marked by a 21.1 percentage-point decline in compression therapy adherence between 30 and 60 days. Primary reasons cited for non-adherence included: discomfort (42.3%), perceived clinical improvement (26.9%), practical difficulties with application (19.2%), and financial constraints (11.6%).

### 3.3 D-dimer trajectories by adherence category

D-dimer trajectories revealed marked differences across adherence categories (Table 2). Patients in the non-adherence group (Group C) demonstrated minimal reduction in D-dimer levels (Δ −0.52 μg/mL; 95% CI: −1.46–0.42; *p* = 0.643), whereas those in the complete adherence group (Group A) showed substantial decreases (Δ −4.87 μg/mL; 95% CI: −5.92 to −3.82; *p* < 0.001). Participants in the partial adherence group (Group B) exhibited intermediate reduction (Δ −3.16 μg/mL; 95% CI: −4.12 to −2.20; *p* < 0.001).

**Table 2:** D-dimer changes from baseline to 90 days by adherence category.

Parameter	Complete adherence (*n* = 40)	Partial adherence (*n* = 44)	Non-adherence (*n* = 26)	*p*-value*
Baseline D-dimer, μg/mL	7.21 ± 4.35	7.68 ± 4.92	7.85 ± 5.14	0.831
90-day D-dimer, μg/mL	2.34 ± 1.28	4.52 ± 2.67	7.33 ± 4.82	<0.001
Absolute change (Δ), μg/mL	−4.87 ± 3.89	−3.16 ± 3.14	−0.52 ± 1.27	<0.001
Percentage change, %	−67.6%	−41.1%	−6.6%	<0.001
Patients with D-dimer >0.5 μg/mL at 90 days, *n* (%)	8 (20.0)	22 (50.0)	21 (80.8)	<0.001

* One-way ANOVA for intergroup comparisons; Values presented as mean ± SD unless otherwise indicated.

The proportion of patients with elevated D-dimer (>0.5 μg/mL) at 90 days varied significantly across adherence categories: 20.0% in Group A, 50.0% in Group B, and 80.8% in Group C (*p* < 0.001). These findings demonstrate a clear dose-response relationship between adherence levels and D-dimer normalization.

### 3.4 Predictors of persistent D-dimer elevation

Multivariable linear regression identified several independent predictors of 90-day D-dimer levels (Table 3). Current smoking emerged as the strongest predictor (*β* = 6.121; 95% CI: 4.52–7.72; *p* < 0.001), followed by non-adherence status (*β* = 4.991; 95% CI: 3.65–6.33; *p* < 0.001) and multiple comorbidities (*β* = 3.171; 95% CI: 2.05–4.29; *p* < 0.001). The regression model explained 62.3% of the variance in 90-day D-dimer levels (R² = 0.623; adjusted R² = 0.601).

**Table 3:** Multivariable predictors of 90-Day D-dimer levels.

Predictor	Unstandardized β	95% CI	Standardized β	t-value	*p*-value
Intercept	3.189	1.52–4.86	-	1.773	0.079
Current smoking	6.121	4.52–7.72	0.494	5.538	<0.001
Non-adherence (vs. complete)	4.991	3.65–6.33	0.430	5.231	<0.001
≥2 Comorbid conditions	3.171	2.05–4.29	0.273	3.639	<0.001
Previous vascular disease	2.658	1.58–3.74	0.230	2.955	0.004
Partial adherence (vs. complete)	2.183	1.21–3.16	0.188	2.766	0.007
Age (per 10-year increment)	0.412	0.08–0.74	0.112	1.982	0.049

Notably, adherence category remained a significant predictor even after adjusting for baseline characteristics, suggesting that management practices exert direct influence on biological risk profiles beyond traditional risk factors.

## 4 Discussion

### 4.1 Limitations and strengths

Before interpreting the findings, several limitations should be acknowledged. First, this was a single-center study conducted in a tertiary hospital in China, which may limit the generalizability of the results to other settings or healthcare systems. Second, adherence was primarily assessed through self-report, which is susceptible to recall and social desirability biases. Third, the follow-up was limited to 90 days, providing insight into the high-risk immediate post-discharge period but not long-term outcomes. Fourth, the primary endpoint was biomarker trajectory; clinical recurrence events were not directly measured, and any implications regarding a “risk window” are inferential based on the association between adherence, D-dimer levels, and established recurrence risk data from the literature. Despite these limitations, the study has notable strengths including its prospective design, comprehensive adherence assessment, standardized biomarker measurement, and novel analysis linking adherence patterns to biological outcomes.

### 4.2 Principal findings and clinical implications

This investigation provides novel evidence establishing a direct biological connection between post-discharge adherence to VTE preventive measures and resolution of hypercoagulability, as quantified through D-dimer normalization. Our findings demonstrate that non-adherent patients experience minimal reduction in D-dimer levels (only 6.6% decrease from baseline), whereas completely adherent patients achieve substantial normalization (67.6% decrease). This ten-fold difference in biomarker response offers compelling evidence that the “post-discharge management gap” has measurable biological consequences with direct implications for recurrence risk.

These observations assume particular significance in light of established evidence linking persistent D-dimer elevation to increased VTE recurrence risk. Multiple prospective studies have demonstrated that elevated D-dimer following anticoagulation cessation is associated with 2–3 fold increased recurrence risk (14, 15). Recent data from the REVERSE cohort further support the prognostic value of serial D-dimer measurements after stopping anticoagulation (16). The PROLONG study revealed that patients with normalized D-dimer levels after anticoagulation withdrawal experienced a 6.2% annual recurrence rate, compared to 15.0% among those with elevated D-dimer (15). Our data suggest that non-adherence may exacerbate this risk by impeding the biological resolution that effective therapy should facilitate.

### 4.3 The 60-Day transition: a critical intervention window

The substantial decline in adherence observed between 30 and 60 days post-discharge (particularly for compression therapy) identifies a specific, actionable intervention point. This temporal pattern aligns with qualitative research identifying the 4–8 week post-discharge period as a phase during which patients transition from “acute recovery” to “chronic management,” often accompanied by reduced clinical support and increased personal responsibility for self-care (42, 43). Understanding the lived experience of patients managing chronic conditions, such as the challenges with self-care routines described in other contexts (44), can further inform support strategies during this transition.

From a clinical perspective, this adherence decline coincides precisely with the highest-risk period for VTE recurrence. Systematic reviews indicate that approximately 40% of recurrences occur within the first three months following the initial event, with peak daily risk during the initial 30 days (45, 46). The adherence deterioration we observed at 60 days thus occurs during a period of substantial residual biological risk, potentially creating a dangerous discordance between patient behavior and underlying pathophysiology.

### 4.4 Smoking as a modifiable determinant of residual risk

The predominant effect of current smoking on D-dimer levels (*β* = 6.121) represents one of our most clinically actionable findings. This effect magnitude substantially exceeds that of traditional risk factors and suggests smoking may represent the most potent modifiable determinant of persistent hypercoagulability in VTE patients. The biological plausibility of this association is well-established, with smoking known to promote endothelial dysfunction, enhance platelet reactivity, increase coagulation factor activity, and establish a pro-inflammatory state (47, 48).

Our observation that 53.8% of non-adherent patients were current smokers (compared to 20.0% of completely adherent patients) suggests clustering of risk factors that may identify a particularly vulnerable subgroup. This finding aligns with research demonstrating that smokers exhibit poorer adherence to medical therapies across multiple conditions (49, 50). Integrating smoking cessation interventions into VTE management protocols may therefore address both a major biological risk factor and a behavioral determinant of poor adherence.

### 4.5 Reconceptualizing VTE: from acute event to chronic risk condition

Our findings challenge the traditional paradigm of VTE as an acute event requiring time-limited treatment. Instead, they support reframing VTE as a chronic high-risk condition characterized by persistent biological vulnerability extending beyond the initial thrombotic episode. This perspective aligns with emerging understanding of VTE pathophysiology as involving chronic endothelial dysfunction, sustained inflammation, and residual hypercoagulability (51, 52). It also resonates with contemporary guideline updates that emphasize structured follow-up and long-term management strategies for DVT patients (20).

This paradigm shift carries important implications for clinical practice and research:

#### 4.5.1 Extended monitoring framework

Rather than focusing exclusively on the acute treatment phase, VTE management should incorporate structured long-term follow-up, with particular attention to the critical 60-day transition period.

#### 4.5.2 Comprehensive risk factor management

Smoking cessation should be integrated as a fundamental component of VTE care, supported by dedicated resources and structured intervention programs.

#### 4.5.3 Biomarker-informed management

D-dimer monitoring provides an objective measure of therapeutic effectiveness and could guide personalized approaches to therapy duration and intensity.

#### 4.5.4 Integrated behavioral-clinical approaches

Addressing adherence requires understanding and intervening upon the behavioral determinants of self-care, particularly during vulnerable transition periods. Enhancing the discharge process through service integration and patient education has been shown to improve outcomes in other contexts and could be applied to VTE care (10, 11).

### 4.6 Future directions

Future research should address the limitations of this study through multicenter designs, extended follow-up durations, objective adherence measures (e.g., electronic monitoring, pharmacy databases), and integration of additional biomarkers reflecting hypercoagulability and inflammation. Particularly valuable would be intervention studies evaluating whether strategies targeting the 60-day adherence transition can improve both adherence and biomarker outcomes. Research should also explore the cost-effectiveness of extended monitoring and support programs in preventing VTE recurrence.

## 5 Conclusion

This investigation establishes a direct biological link between the post-discharge “management gap” in VTE care and persistent hypercoagulability, as evidenced by minimal D-dimer reduction among non-adherent patients. Our findings support reconceptualizing VTE as a chronic high-risk condition necessitating structured long-term management rather than an acute event with time-limited treatment.

Three principal implications emerge:

The 60-day post-discharge period constitutes a critical intervention window requiring enhanced support to prevent adherence deterioration during this high-risk phase.
Smoking cessation should be integrated as a fundamental component of VTE management, addressing both a major biological risk factor and a determinant of poor adherence.
D-dimer monitoring provides objective evidence of management effectiveness and could inform personalized approaches to therapy duration and intensity.

Looking forward, VTE care must transition from a paradigm of “acute treatment completion” to one of “chronic risk control,” with particular emphasis on the vulnerable hospital-to-community transition. By addressing both behavioral (adherence) and biological (hypercoagulability) dimensions of post-discharge management, we can potentially reduce the substantial burden of VTE recurrence and its associated morbidity and mortality.

## Data Availability

The original contributions presented in the study are included in the article/Supplementary Material, further inquiries can be directed to the corresponding author.

## Appendix

### Appendix 1: patient adherence scoring and group classification matrix

This matrix provides the operationalized definitions for categorizing patients into Complete Adherence (Group A), Partial Adherence (Group B), and Non-Adherence (Group C) groups, as described in Section 2.3. Classification is based on data collected over the 90-day post-discharge follow-up period.

**Table:**

Domain & metric	Operational definition & scoring criteria
A. Anticoagulation therapy adherence	
1. Treatment completion	(1) Met: Completed the full prescribed 90-day course without premature discontinuation. (Score: 2) (2) Not Met: Discontinued therapy prematurely (<90 days). (Score: 0)
2. Self-reported adherence behavior	(1) No Intentional Deviation: Patient reported no deliberate omissions or self-directed dosage modifications. (Score: 1) (2) Intentional Deviation Reported: Patient admitted to such behaviors. (Score: 0)
B. Compression therapy adherence	
3. Elastic stocking use	(1) High: Used stockings ≥8 h/day on ≥80% of days. (Score: 2) (2) Partial: Used stockings ≥8 h/day on 50–79% of days. (Score: 1) (3) Low/Nil: Used on <50% of days. (Score: 0)
C. Composite group classification rules	
Group A (complete adherence)	Must satisfy ALL: (1) Anticoagulation: Completed (Score: 2)AND No Intentional Deviation (Score: 1). (2) Compression: High Use (Score: 2). → Total Score = 5
Group B (partial adherence)	Characterized by ANY of the following profiles: (1) Profile 1 (Anticoagulation Adequate, Compression Inadequate): Anticoagulation Completed (Score: 2) with No Intentional Deviation (1), but Compression = Partial or Low (Score 1 or 0). (2) Profile 2 (Compression Adequate, Anticoagulation Inadequate): Compression High (Score: 2), but Anticoagulation Not Completed (Score: 0). (3) Profile 3 (Minor Deviations in Both): Both therapies partially adhered to (e.g., Anticoagulation Completed but with Intentional Deviation [resulting in score 1], Compression Partial （Score: 1）). → Total Score typically 2–4
Group C (non-adherence)	Defined by meeting BOTH of the following conditions: (1) Anticoagulation Failure: Treatment Not Completed (Score: 0) OR Intentional Deviation Reported (Score: 0). (2) AND (3) Compression Failure: Compression Use = Low/Nil (Score: 0). → Total Score typically ≤ 2

Notes on Classification Procedure:
Initial screening is performed based on the two core criteria: “Anticoagulation Course Completion” and “Elastic Stocking Use on ≥80% of days.” Final classification integrates self-reported adherence behavior and the degree of compression therapy use. The “Partial Adherence” group encompasses a spectrum from “adequate adherence to one core therapy” to “minor deviations in both.” This matrix ensures clinical relevance and reproducibility by outlining typical profiles.
